# Liquid chromatography and differential mobility spectrometry—data-independent mass spectrometry for comprehensive multidimensional separations in metabolomics

**DOI:** 10.1007/s00216-023-04602-0

**Published:** 2023-02-23

**Authors:** Lysi Ekmekciu, Gérard Hopfgartner

**Affiliations:** Life Sciences Mass Spectrometry, Department of Inorganic and Analytical Chemistry, University of Geneva, 24 Quai Ernest Ansermet, 1211 Geneva 4, Switzerland

**Keywords:** Metabolomics, Differential mobility spectrometry, Liquid chromatography–mass spectrometry, SWATH-MS, Human urine, Toxicology

## Abstract

The benefits of combining drift time ion mobility (DTIMS) with liquid chromatography–high-resolution mass spectrometry (HRMS) have been reported for metabolomics but the use of differential time mobility spectrometry (DMS) is less obvious due to the need for rapid scanning of the DMS cell. Drift DTIMS provides additional precursor ion selectivity and collisional cross-section information but the separation resolution between analytes remains cell- and component-dependent. With DMS, the addition of 2-propanol modifier can improve the selectivity but on cost of analyte MS response. In the present work, we investigate the liquid chromatography–mass spectrometry (LC–MS) analysis of a mix of 50 analytes, representative for urine and plasma metabolites, using scanning DMS with the single modifiers cyclohexane (Ch), toluene (Tol), acetonitrile (ACN), ethanol (EtOH), and 2-propanol (IPA), and a binary modifier mixture (cyclohexane/2-propanol) with emphasis on selectivity and signal sensitivity. 1.5% IPA in the N_2_ stream was found to suppress the signal of 50% of the analytes which could be partially recovered with the use of IPA to 0.05% as a Ch/IPA mixture. The potential to use the separation voltage/compensation voltage/modifier (SV/CoV/Mod) feature as an additional analyte identifier for qualitative analysis is also presented and applied to a data-independent LCxDMS-SWATH-MS workflow for the analysis of endogenous metabolites and drugs of abuse in human urine samples from traffic control.

**Graphical Abstract:**

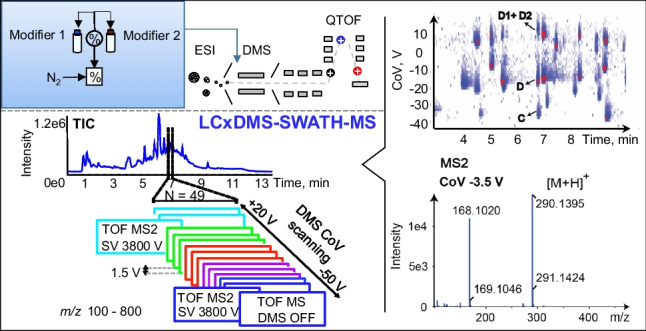

**Supplementary Information:**

The online version contains supplementary material available at 10.1007/s00216-023-04602-0.

## Introduction

Metabolomics investigations provide a molecular read-out of low molecular weight compounds (LMWC) in biological samples such as plasma, urine, or tissues and have gained interest in clinical studies to describe patient’s health or disease status [1, 2]. Liquid chromatography coupled to mass spectrometry (LC–MS) has become the analytical strategy of choice for targeted and untargeted analysis but has to deal with a large diversity of endogenous and exogenous compounds (e.g., lipids, amino acids, hormones, nucleotides, pharmaceutical, pesticides) having diverse chemical properties (MW, pKa, Log(p)) [3]. In LC–MS untargeted approach, the scope of analysis is to identify and quantify as many metabolites as possible in large sample sets (e.g., 100–1000 samples) with analysis time, typically less than 30 min. Reversed-phase liquid chromatography (RPLC) and hydrophilic interaction liquid chromatography (HILIC) offer different chromatography selectivity but suffer from limited separation power even in ultra-high-performance mode and many analytes co-elute and separation of isomeric/isobaric metabolites remains challenging. LC peak capacity can be significantly improved by applying comprehensive two-dimensional liquid chromatography (LCxLC) by combining different separation modes including partition, adsorption, size exclusion, ion exchange, or affinity chromatography [4, 5]. Nevertheless, optimization of generic LCxLC separation is time-consuming due to several sample-independent parameters such as column dimensions, particle sizes, flow rates, and mobile-phase compatibility. Over the last years, the use of ion mobility spectrometry (IMS) [6], which exploits the mobility of gas-phase ions, has gained interest in bioanalysis in combination with liquid chromatography either as a selectivity filter with differential mobility spectrometry (DMS), also often referred as field-asymmetric waveform ion mobility spectrometry (FAIMS), or as a separate dimension with drift time ion mobility spectrometry (DTIMS) or trapped ion mobility spectrometry (TIMS). While DTIMS and TIMS exploit the instantaneous mobility of gas-phase ions in the presence of a constant electric field, DMS takes advantage of different mobilities of gas-phase ions when in the presence of low and high electric fields. The DMS cell is placed at atmospheric pressure between the ion source and the vacuum interface, and an asymmetric electric field, the separation voltage (SV), is applied between two planar electrodes perpendicular to the movement of the ions, and to correct the ion trajectory, a compensation voltage (CoV) is applied [7]. The DMS can be operated in filter mode with a fixed CoV or in scanning mode by stepping the CoV over a certain range. The addition of polar or apolar modifiers (e.g., 2-propanol, toluene) in the nitrogen stream forms clusters with the charged ions, resulting in a significant shift towards positive or negative CoV values, and can be applied to tune the selectivity in particular for the separation of co-eluting isomeric compounds [8].

Multidimensional LC-IMS-MS, mostly based on DTIMS combined to quadrupole time-of-flight mass spectrometry (QqTOF), has been demonstrated to be useful in untargeted metabolomics to improve metabolome coverage, data quality, analysis throughput, and isomer separation [9, 10]. Small molecules can be characterized, in addition to retention time and *m/z*, by IMS drift times and collision cross section (CCS). Wernisch et al. [11] reported a study including 800 metabolites where they investigated the orthogonality of liquid chromatograph and generic DMS using 1.5% 2-propanol as a modifier (SelexION, Sciex). They observed the best orthogonality to DMS with hydrophilic interaction liquid chromatography (HILIC) based on retention time and CoV values and applied it to the analysis of plasma extracts of 10 CKD patients [12]. However, the DMS modifier, and in particular 2-propanol, can suppress the analyte signal [7]. Ruskic et al. [7] proposed to use binary modifiers mixture at various concentrations (e.g., cyclohexane/2-propanol) with untargeted LCxDMS-MS to optimize signal intensity, CoV range, and isomer separation performance. HILIC was also combined with a fast-scanning miniaturized FAIMS cell (Owlstone) mounted in front of a QqTOF mass spectrometer operate at 20 scan/second for the untargeted metabolomic analysis of urine samples using in-source collision-induced dissociation.

To investigate metabolomics samples with co-elution of multiple compounds, IM-MS with LC separation data-dependent acquisition (DDA) and data-independent acquisition (DIA) (e.g., MS Everything, diaPASEF) MS/MS techniques are applied, providing four dimensions of separation including retention time, collision cross section (for IMS) or compensation voltage (for DMS), MS1, and MS/MS information [13, 14]. SWATH is another DIA approach which uses multiple Q1 selection windows, typically of 25 Da, and product ions are analyzed in a second stage [15]. The major benefit of DIA is the possibility of re-interrogating the sample post-acquisition for qualitative or quantitative analysis. The combination of scanning DMS with a large SWATH window has been reported by Sosnowski et al. [16] and enabled the differentiation of isobaric signals from illicit drugs with a large dynamic range and enhanced the information contained in these pills by acquiring MS2-level information.

In this paper, we describe the LC–MS analysis of a mix of 50 analytes, representative of urine and plasma metabolites, using scanning DMS with single modifiers and binary modifiers with an emphasis on selectivity and signal sensitivity. The potential to use SV/CoV/mod information as an additional analyte identifier is also discussed and applied for the LCxDMS-SWATH-MS analysis of endogenous metabolites and drugs of abuse in human urine samples from traffic control.

## Materials and methods

### Chemicals

Standard compounds for mix 50 were purchased from different suppliers (Table S1). Acetaminophen-D4 was purchased from Toronto Research Chemical and naproxen-D3 from Sigma-Aldrich. The following solvents were used as DMS modifiers and HPLC mobile phases: HPLC-grade cyclohexane (Ch), toluene (Tol), methanol (MeOH) from Carl Roth (Switzerland); acetonitrile (ACN), ethanol (EtOH), 2-propanol (IPA) from VWR (Darmstadt, Germany); and water was from Huberlab (Aesch, Switzerland). Formic acid (FA) was provided by Merck (Darmstadt, Germany) and ammonium formate from Honeywell Fluka.

### Sample preparation

#### Mix 50

Individual stock solutions of 1 mg/ml were prepared in methanol, water, ethanol, or methanol/water (1:1 v/v) or ethanol/water (1:1 v/v). Mix 50 was obtained by a collection of all standards with corresponding taken volumes as described in Table S1 and afterwards were evaporated using N_2_.

#### Human urine samples

Urine samples, which tested positive for THC and/or cocaine, were provided by the Institute of Forensic Medicine from the University of Bern, Switzerland, and were collected during roadside drug testing. All samples were stored at − 20 °C. For LC experiments, 20 µl of a mixture of two internal standards (acetaminophen-D4 and naproxen-D3) at a concentration of 50 and 500 ng/µl was added to 80 µl of urine samples.

#### Liquid chromatography

Reverse-phase separation was performed using a Nexera UHPLC (Shimadzu Corporation, Kyoto, Japan) composed of one degasser DGU-20A 5R, pump LC-30AD, autosampler SIL-30AC, and oven CTO-30A. Analytes were separated in a XSelect HSS T3 (150 mm × 2.1 mm I.D, particle size of 2.5 µm) analytical column at 40 °C, using 5 mM ammonium formate and 0.1% formic acid (v/v) in water as eluent A, and 5 mM ammonium formate and 0.1% formic acid (v/v) in methanol as eluent B. The gradient at a flow rate of 0.3 ml/min was as follows: 5% B from 0 to 1 min, then increased from 1 to 21 min to 90% B, constant from 21 to 25 min to 90% B, and decreased from 26 to 30 min to 5% B. The gradient was reduced for urine samples to 5% B from 0 to 1 min, then increased from 1 to 5 min to 90% B, constant from 5 to 9 min to 90% B, and decreased from 10 to 14 min to 5% B. Injection volume for mix 50 was 5 µl and for urine samples 10 µl.

#### DMS-time-of-flight mass spectrometry (TOFMS)

A quadrupole time-of-flight (QTOF) mass spectrometer (TTOF 6600 + , Sciex, Concord, ON, Canada) was equipped with a differential ion mobility device (SelexION, Sciex). A DuoSpray ion source was used in positive mode at 4500 V. The temperature of the ion source was set to 350 °C, the nebulizer gas (GS1) was set to 30 psi, the drying gas (GS2) was set to 30 psi, and the curtain gas was set to 15 psi.

For DMS experiments, the following settings were used: DMS cell temperature 150 °C, separation voltage for all experiments 3800 V except for modifier toluene which is 4000 V, compensation voltage (CoV) was ramped from − 15 to + 30 V by steps of 1 V for N_2_ experiments and − 50 to + 20 V by steps of 1 V for all modifiers, DMS offset (DMO) was set to − 3 V for N_2_ experiments and to + 30 V for experiments with modifiers for CoV lower than − 20 V.

For the delivery of binary modifiers, the SelexION single-channel pump was replaced by a binary HPLC pump (Agilent 1100, Agilent Technologies, Germany). DMS experiments were conducted using nitrogen or 1.5% mole ratio chemical modifiers or binary modifiers (1.5% mole ratio of 97:3 v/v cyclohexane: 2-propanol) in nitrogen corresponding to 0.05% mole ratio 2-propanol. Modifiers were introduced in isocratic mode at 1.5% Ch in N_2_, mole ratio of 284 µl/min; 1.5% ACN in N_2_, mole ratio of 138 µl/min; 1.5% EtOH in N_2_, mole ratio of 154 µl/min; 1.5% IPA in N_2_, mole ratio of 200 µl/min; 1.5% Tol in N_2_, mole ratio of 280 µl/min; and 0.05% IPA in N_2_, mole ratio of 284 µl/min.

#### LCxDMS-SWATH-MS experiments

LCxDMS-MS analyses were performed using SWATH acquisition in positive ion mode ESI for urine samples from traffic control with a total cycle time of 1354 ms. MS acquisition was controlled by Analyst version 1.6. MS1 data were acquired from *m/z* 100 to 800 with an accumulation time of 50 ms. For MS1 experiments, the CoV value was set to 0 V and SV to 500 V. Followed by MS2 performed with 48 CoV-SWATH Q1 windows (*m/z* 100–800) and MS2 TOF range *m/z* 100–800 with an accumulation time of 25 ms. For SWATH experiments, the DMS SV was 3800 V and the CoV was ramped from − 50 to + 20 V by steps of 1.5 V. The collision energy (CE) was set to 10 V for MS1 and to induce fragmentation to 25 eV with a collision energy spread (CES) of 15 eV for MS2 experiments. Declustering potential (DP) was set to 80 V for all experiments and a settling time of 50 ms only for the first MS2 experiment. A binary mixture of modifiers 0.05% mole ratio IPA was introduced to the DMS cell pumped at an isocratic flow rate of 284 µl/min, modifier density of 0.779 g/ml, and modifier molecular weight of 84.16 g/mol.

#### Data processing

The data were processed with PeakView (version 2.2) and MasterView software (Sciex). Library searches were performed with the following libraries: HRAM Forensics_v1.1, LSMS ExpLib v1, and MSMS Public Pos V15. A prototype plugin DMSInspector v1.0 (Sciex) was used for compound identification and CoV determination of each analyte.

## Results and discussion

The addition of IMS in a LC–MS workflow offers either in the case of DTIMS better precursor ion selection for MS/MS or the CCS value can be used as a compound identifier to improve identification. Nevertheless, the resolution depends on the hardware and the CCS is analyte-dependent. In the case of DMS, improved precursor selection is also possible, but the addition of modifiers opens the tuning of the separation selectivity while 2-propanol (IPA) is the most popular one as it generates the highest negative CoV shifts. CoV values with IPA were generated for a set of 800 metabolites in positive and negative ionization modes, but the effect of IPA on MS response was not investigated. [11] In the present work, we evaluate first the MS response and the selectivity for DMS modifiers (IPA, cyclohexane, toluene, acetonitrile, and ethanol) as well as for binary mixture DMS modifiers (cyclohexane with IPA).

### MS response using different modifiers

**Fig. 1:**
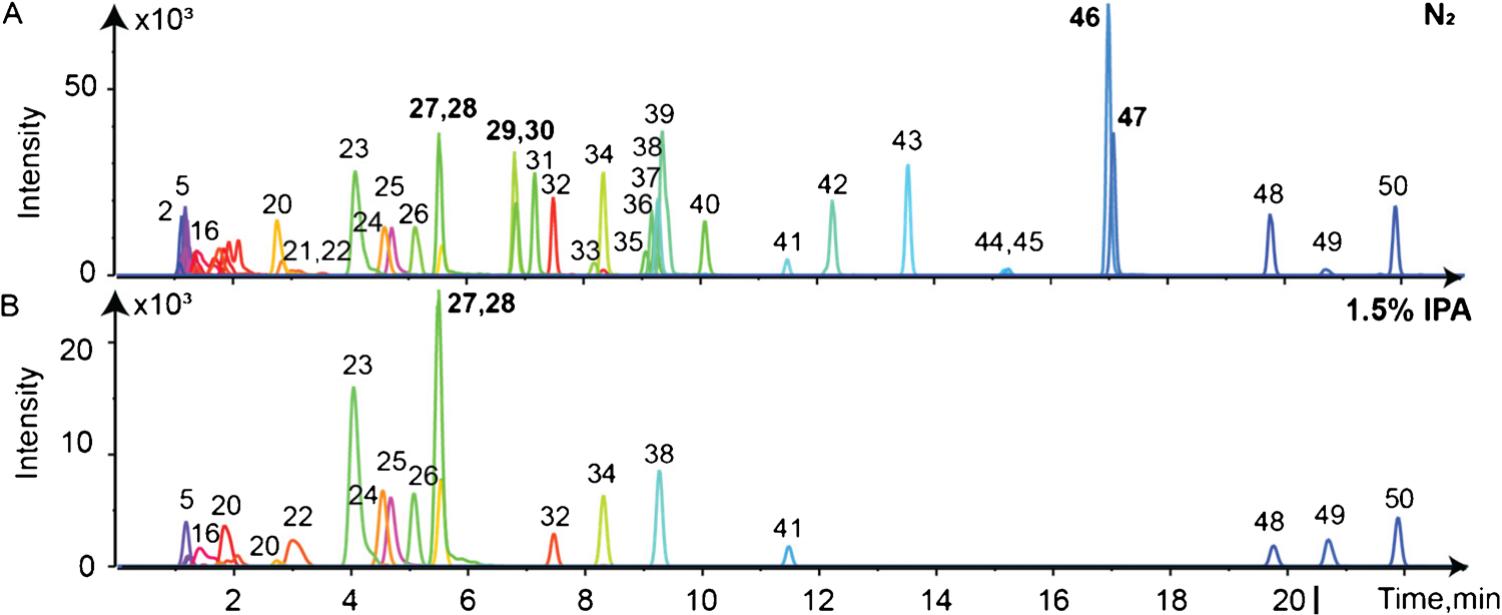
LCxDMS-MS analysis, XIC for mix 50 **A** pure nitrogen, and **B** 1.5% mole ratio IPA in positive ionization mode. 27 ethenodeoxyadenosine, 28 3-chlorotyrosine, 29 pantothenic acid, 30 acetaminophen, 46 phloretin, and 47 cortisone (for detailed peak assignment, see Table S2)

The LC-DMS-MS analysis with N_2_ for a test mix of 50 analytes using a 20-min gradient, representative of urine metabolites, is presented in Fig. 1A and showed several analyte co-elution: L-glutamine (*m/z* 147.0764), homo-L-arginine (*m/z* 189.1346), glycerophosphocholine (*m/z* 258.1101) at RT = 1.2 min, 3-chlorotyrosine (*m/z* 216.0422) and ethenodeoxyadenosine (*m/z* 276.1091) at RT = 5.3 min, acetaminophen (*m/z* 152.0706) and pantothenic acid (*m/z* 220.1180) at RT = 6.8 min, phloretin (*m/z* 275.0914) and cortisone (*m/z* 361.2009) at RT = 17.0 min are a few examples. As these analytes have different precursor ion masses, they can be differentiated by HRMS and targeted MS/MS. However, in DIA acquisition mode, MS2 spectra are generally composite spectra in particular as many metabolites form in-source fragments (water and ammonia loss), metal adduct ions, or multimers challenging analyte identification [17]. Figure 1 compares the extracted ion current of LCxDMS-MS analyses with N_2_ and 1.5% IPA for mix 50. Compared to N_2_ in DMS, the addition of 1.5% IPA resulted in a signal loss of 25 analytes out of 50 analytes (Table S2) for 1.5% IPA as a modifier. The signal decrease of suppression of the 25 analyte is mainly due to gas-phase reactions with IPA, and a strong hydrogen bond formed between IPA. The extracted ion current of LCxDMS-MS analyses for N_2_ and all six modifiers is presented in Figure S1.

**Fig. 2:**
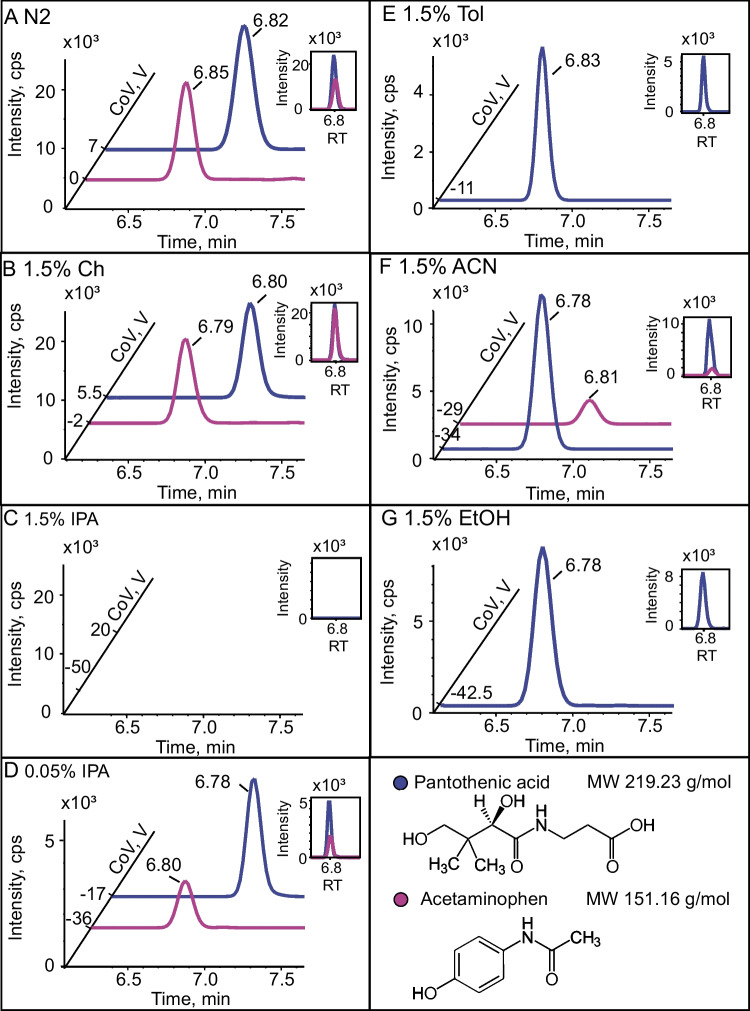
Ionograms of co-eluting pantothenic acid and acetaminophen, blue and magenta respectively, from mix 50 analytes obtained from LCxDMS-MS experiments with modifiers **A** nitrogen (N_2_), **B** 1.5% cyclohexane (Ch), **C** 1.5% 2-propanol (IPA), **D** 0.05% 2-propanol, **E** 1.5% toluene (Tol), **F** 1.5% acetonitrile (ACN), and **G** 1.5% ethanol (EtOH)

Pantothenic acid and acetaminophen are two representative analytes which are completely suppressed with 1.5% IPA and ionograms with different modifiers are shown in Fig. 2. In the DMS dimension with N_2_, pantothenic acid and acetaminophen are detected at CoV + 7 V and 0 V with intensities of 30e3 cps and 10e3 cps, respectively (Fig. 2A). CoV peak width at the base is typically 7 to 10 V. For 1.5% mole ratio cyclohexane (Ch), which is a non-clustering modifier, similar CoV to N_2_ experiments are observed at + 5.5 V and − 2 V respectively with both intensities of 30e3 cps. There is no benefit to using hydrocarbons such as cyclohexane in DMS as a single modifier but as a mixture and its major role is to quickly deliver a small percentage of a clustering modifier without the need for cell re-equilibration and to obtain reproducible CoVs [7]. While IPA and EtOH are miscible with Ch, water, methanol, and acetonitrile are not miscible with Ch. To investigate the effect of a lower percentage (< 1.5% mole ratio) of IPA, a binary mixture of Ch:IPA at 97:3 *v:v* ratio resulting in a final concentration of 0.05% IPA was introduced to the DMS cell. Pantothenic acid and acetaminophen are detected at CoV − 17 V and − 36 V respectively with a sixfold (for pantothenic acid) and fivefold (for acetaminophen) decrease in sensitivity compared to N_2_ experiments. The use of a lower concentration of IPA enables good separation without completely losing the signal. With toluene (Tol), an apolar aromatic solvent (Fig. 2E), pantothenic acid was detected at CoV − 11 V with a sixfold decreased intensity compared to the N_2_ experiment, and acetaminophen was not detected. This is probably due to the strong π-π interaction created between the two aromatic cycles of acetaminophen and toluene. With acetonitrile (ACN) as a modifier (Fig. 2F), both co-eluting analytes, pantothenic acid and acetaminophen, were separated at CoV − 34 V and − 29 V respectively with intensities of threefold (for pantothenic acid) and fivefold (for acetaminophen) lower compared to N_2_. Finally, with ethanol (EtOH) (Fig. 2G), another protic modifier as IPA, pantothenic acid is detected at CoV − 42.5 V with an intensity of 3.8-fold lower compared to N_2_, and acetaminophen was not detected.

**Fig. 3:**
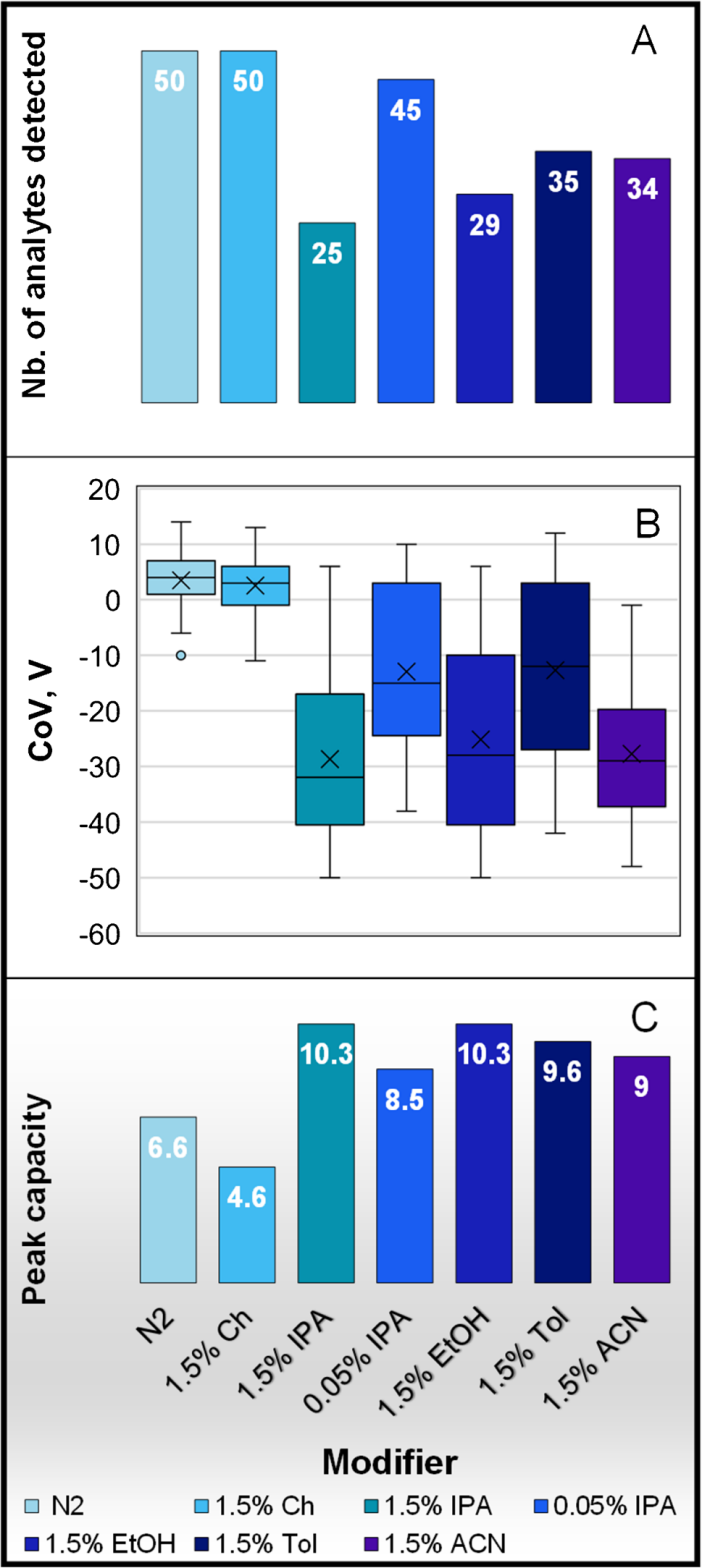
LCxDMS/MS experiments for mix 50 analytes with pure nitrogen(N2), modifiers (1.5% mole ratio): cyclohexane (Ch), 2-propanol (IPA), ethanol (EtOH), toluene (Tol), acetonitrile (ACN), and binary modifier 0.05% IPA **A** comparison of sensitivity representing the detected analytes, **B** CoV shift range for each modifier, and **C** peak capacity for each modifier

To summarize, for mix 50 analytes (Fig. 3A), 50 analytes were detected for pure N_2_ and Ch (1.5% mole ratio), 45 analytes for IPA (0.05% mole ratio), 35 analytes for Tol (1.5% mole ratio), 34 analytes for ACN (1.5% mole ratio), 29 analytes for EtOH (1.5% mole ratio), and least with 25 analytes for IPA (1.5% mole ratio) representing 100%, 90%, 70%, 68%, 58%, and 50% respectively. Finally, the binary modifier 0.05% mole ratio IPA was found to be a good generic compromise with regard to analyte peak intensities (Table S3).

### DMS CoV shifts and peak capacity using different modifiers

By adding modifiers in the N_2_ stream, the charged analytes can form clusters. With the clustering − declustering mechanism, the effective CCS of an analyte ion increases with the modifier at a low field compared to pure nitrogen. The ion behavior in DMS is more related to the physicochemical properties of the analyte and modifier than to the ion’s mass and CCS alone [8]. By using different modifiers, different interactions are created with the analytes present in the sample; therefore, we observe various CoV shifts. Peak capacity measures the spread of peaks in the CoV space divided by the average peak width at half height (PWHH) [18] and was determined for each modifier (1.5% mole ratio Ch, IPA, ACN, Tol, and EtOH as well as 0.05% mole ratio IPA) (Fig. 3C and Table S2 for a summary of CoV). With N_2_, CoV shifts between − 10 and + 14 V with PWHH of 3.6 V, corresponding to a peak capacity of 6.6. For 1.5% mole ratio Ch, CoV shifts between − 11 and + 13 V with PWHH of 5.2 V, corresponding to a peak capacity of 4.6. For 1.5% mole ratio IPA, two times larger CoV shift (compared with N_2_) was observed with a CoV shift between − 50 and + 6 V with PWHH of 5.4 V, corresponding to a peak capacity of 10.3, but a significant loss in response to 50% of analytes. For 0.05% IPA, two times larger CoV shift (compared with N_2_) was observed with a CoV shift between − 38 and + 10 V with PWHH of 5.6 V, corresponding to a peak capacity of 8.5, without sacrificing the response of analytes. For 1.5% mole ratio Tol, ACN, and EtOH, a similar CoV range of 50 V was observed with PWHH of about 5 V (Fig. 3B). 0.05% mole ratio IPA was decided as the generic modifier which provides a good peak capacity compared to the other modifiers without sacrificing sensitivity.

### DMS selectivity tuning using different modifiers

With the binary 0.05% IPA modifier mix, compared to N_2_, pantothenic acid and acetaminophen show a negative CoV shift (Fig. 2D) at − 17 V and at − 36 V compared to + 7 V and 0 V, respectively in N_2_ conditions. For 1.5% mole ratio ACN (Fig. 2F), inversion of selectivity for the two analytes was observed with CoV for pantothenic acid at − 34 V and acetaminophen at − 29 V.

**Fig. 4:**
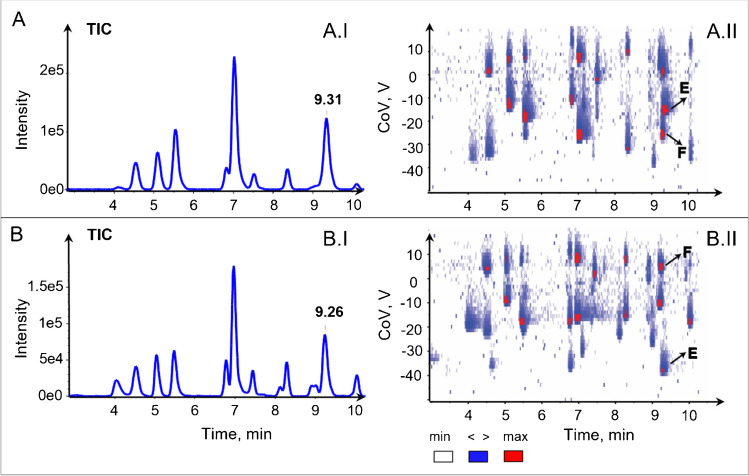
Selectivity inversion represented by heat map of LCxDMS/MS experiments was shown for mix 50 analytes from 3 to 10 min with modifiers **A** 1.5% toluene, **B** 0.05% 2-propanol. The inversed selectivity was observed for (E) [5'-methylthioadenosine + H]^+^ and (F) [quinaldic acid + H]^+^

Another example of selectivity inversion between 1.5% mole ratio Tol and 0.05% mole ratio IPA is represented in Fig. 4 for 5′-methylthioadenosine and quinaldic acid which are co-eluting at RT = 9.31 min with modifier 1.5% mole ratio toluene (Fig. 4A.I), while the separation at CoV − 26 V and − 16 V, respectively, is showed by the heat map (Fig. 4A.II). The selectivity inversion can be explained by the thermochemistry of the cluster formation between an ion and a neutral molecule which plays a decisive role in CoV shift and therefore selectivity between two analytes [8]. Compared to DTIMS where only the resolution can be optimized, DMS offers the possibility to tune the selectivity of the DMS separation based on the properties of the modifiers and the type of interaction with the analyte. This is of interest in the multidimensional separation of complex mixtures by LCxDMS where different types of selectivity can be combined in the same way as for LCxLC without any hardware change or optimization.

**Fig. 5:**
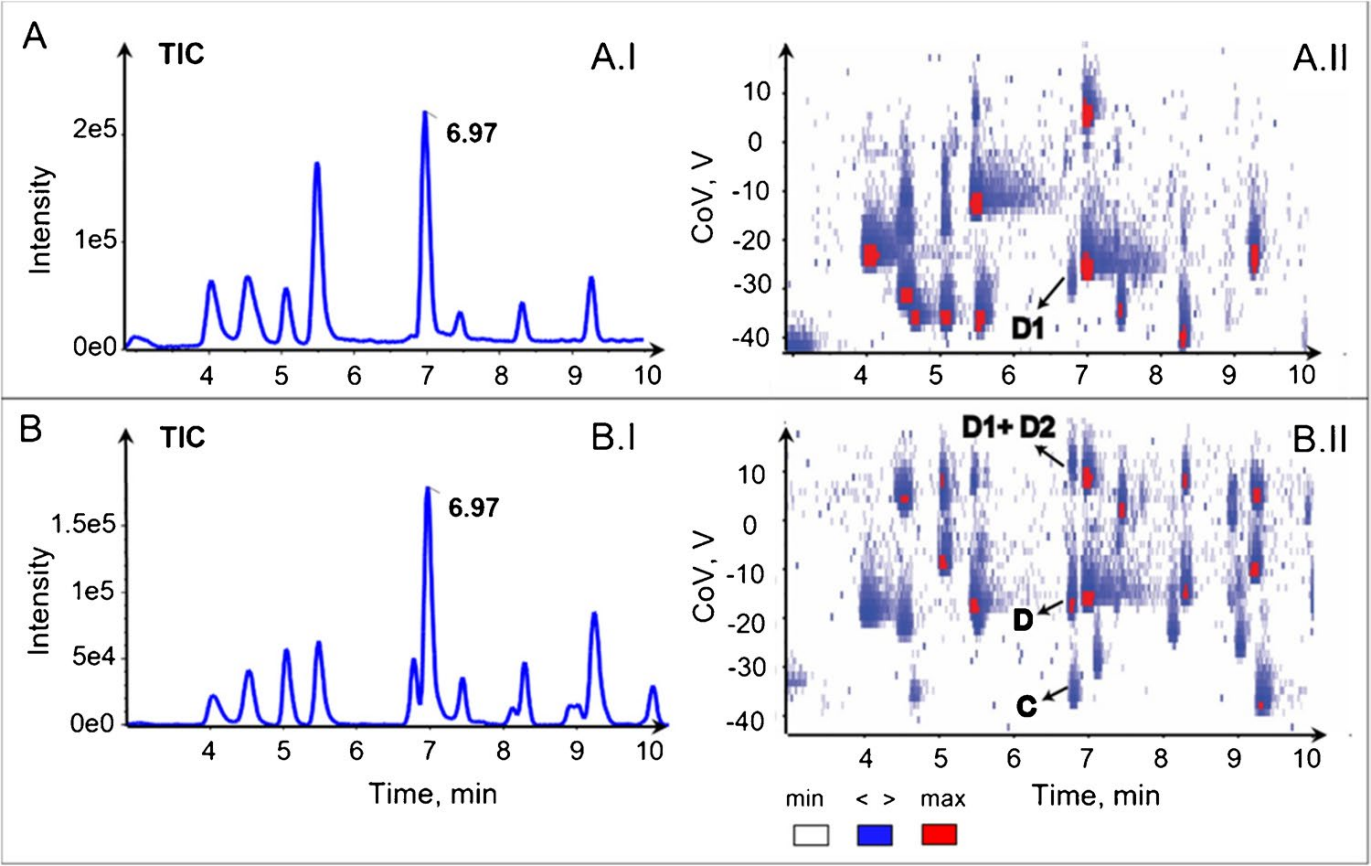
Adduct separation represented by heat map of LCxDMS/MS analysis of mix 50 analytes from 3 to 10 min was shown with modifiers **A** 1.5% 2-propanol, **B** 0.05% 2-propanol for analytes (D) [pantothenic acid + H]^+^, (D1) [pantothenic acid + Na]^+^, (D2) [2pantothenic acid + Na]^+^, (C) [acetaminophen + H]^+^

In LC–MS analysis of biological samples such as plasma or urine, the correct identification and the accurate quantification of metabolites remains challenging, due to the presence of metal adducts (Na, K, Ca, Ba, etc.) and multimers in the spectrum [19]. Multimers and adducts have different mobilities to the protonated analytes as illustrated in Fig. 5 which compared DMS with 1.5% IPA and with binary modifier 0.05% IPA, respectively. At RT of 6.97 min, the sodium adduct of pantothenic acid (D1) was detected at CoV − 29 V (Fig. 5A.II), but the protonated pantothenic acid was not observed as previously described. This nicely illustrated the need for adduct annotation tools also with IMS for analyte identification. When the modifier 0.05% IPA was used, acetaminophen (C) and pantothenic acid (D) were separated in the DMS dimension at CoV − 36 V and − 17 V, respectively, but also the sodium adduct of pantothenic acid dimer (D2) from sodium adduct of pantothenic acid (D1) at CoV + 11 V could be observed (Fig. 5B.II).

### Characterization of analytes by SV/CoV

Drift time ion mobility measures the collision cross section (CCS) of an ion, related to its chemical structure, size, shape, and charge, and since CCS measurements have high reproducibility, independent of LC conditions, CCS libraries are built for analyte identification and/or confirmation [20]. In DMS, the measurement of CCS value is not possible, but for a modifier and given SV given the CoV is reproducible (± 2 V) within a laboratory frame and even between different instruments given similar settings are used (data not shown). Therefore, the combination SV/CoV and modifier could be used as an additional analyte identifier independent from LC conditions.

For the test mix 50 with different modifiers, SV/CoV reference tables were created for all detected analytes (Table S2) and can be used for searching these analytes, in complex mixtures such as human urine with modified LC conditions.

## LCxDMS-SWATH-MS: analysis of human urine samples

**Fig. 6:**
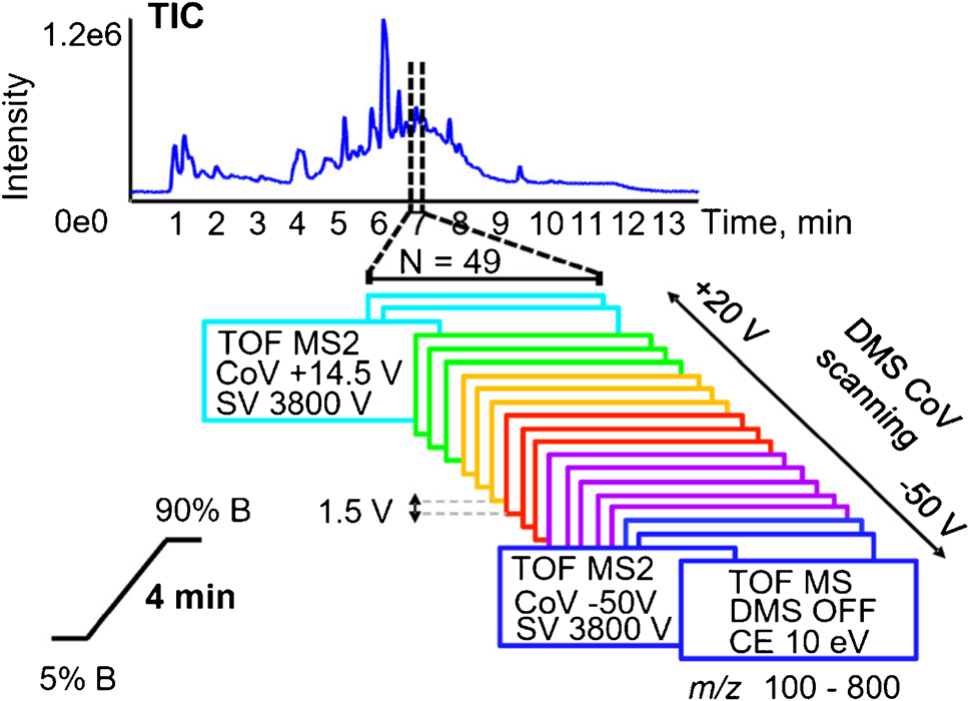
LCxDMS-SWATH-MS acquisition workflow in positive ionization mode for the analysis of urine samples with modifier 0.05% mole ratio IPA. The MS method is built of 1 TOF MS followed by 48 MS2 experiments with a total cycle time of about 1.4 s. Scanning DMS with CoV from − 50 to + 20 V, by 1.5 V steps are applied to MS2 experiments. SV was of 3800 V

Feature detection in untargeted metabolomic workflow generally relies on analyte retention time, accurate mass, intensity, and more recently on CCS. The identification of analyte characterization requires MS/MS spectra. DIA workflows such as MSE or SWATH are of particular interest for untargeted LC–MS as all precursors are fragmented and the sample can be re-interrogated post-acquisition analysis but show limitation of identification and quantification in particular when short LC gradient for higher throughput is used. Contrary to MS^Everthing^, SWATH offers the possibility to use variable Q1 windows from 1 m*/z* to several hundred *m/z* [21]. As illustrated in Fig. 6, we implemented a workflow formed by 47 experiments integrating scanning DMS with SWATH acquisition. The first experiment provides MS1 data where the DMS cell is operated in transparent mode. In the 48 additional experiments, the DMS is operated in scanning mode with CoV steps of 1.5 V covering a CoV range from − 50 to + 20 V. The total cycle time of 1.4 s is compatible with the LC timescale. The collision cell energy range was from 10 to 40 eV to maintain some residual precursor ions in the MS2 spectrum.

**Fig. 7:**
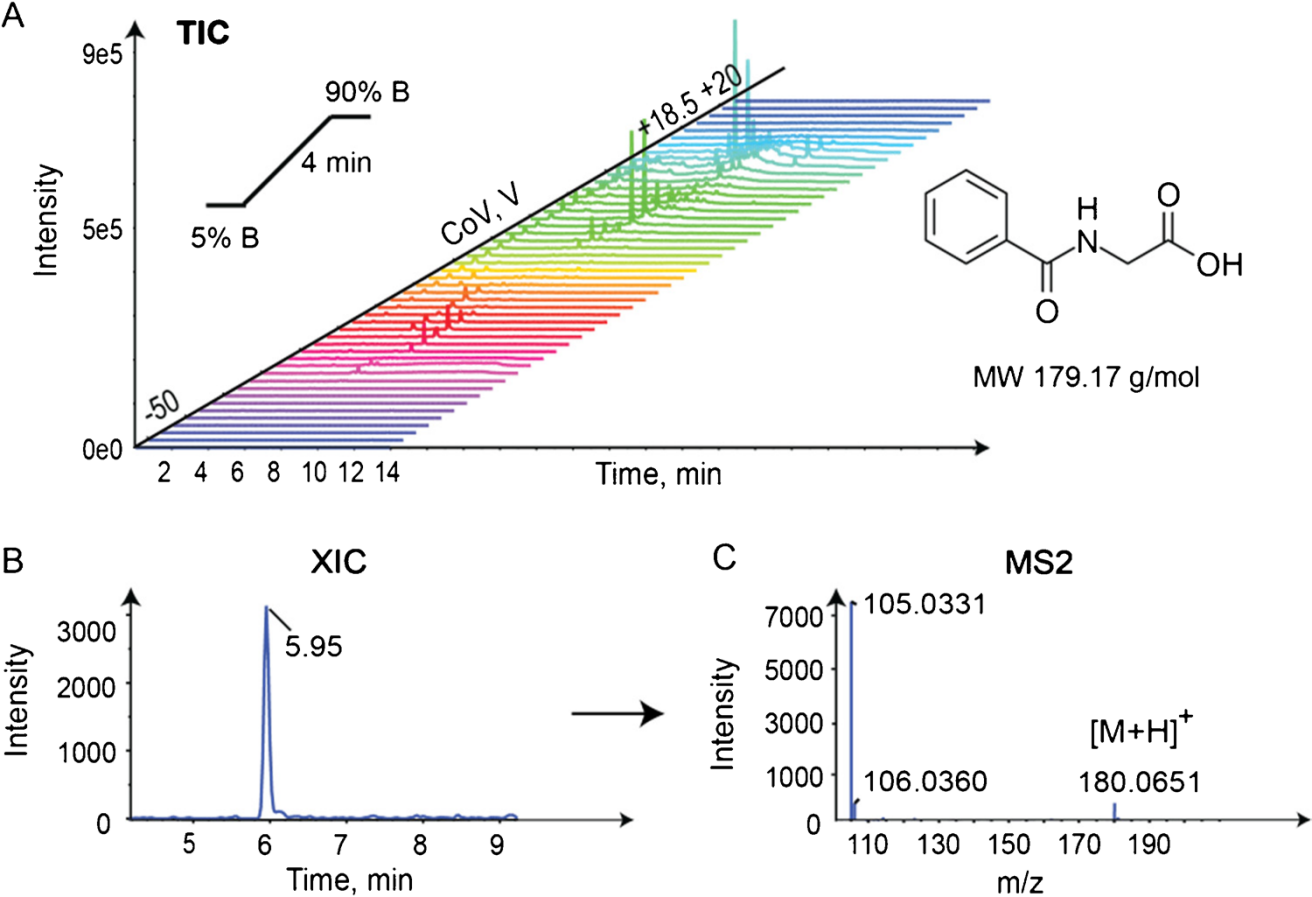
**A** TIC of urine sample for LCxDMS/SWATH-MS experiment with LC gradient of 4 min and CoV scanning from − 50 to + 20 V, by 1.5 V steps with modifier 0.05% mole ratio IPA, **B** XIC of hippuric acid at CoV − 25 V, **C** MS2 spectrum of hippuric acid at CoV − 25 V (expected at SV/CoV 3800 V/ − 26 V from mix 50)

In this work, urine samples from traffic control positive to cocaine and/or THC were analyzed using LCxDMS-SWATH-MS with a short 4-min LC gradient and 0.05% IPA as a modifier and a representative sample is presented in Fig. 7A. In the first step, hippuric acid was detected at RT = 5.95 min and CoV − 25 V (Fig. 7B) and MS2 spectra from SWATH acquisition is retrieved at the same CoV − 25 V, having 1 V of difference compared with expected reference CoV from mix 50 which is within tolerance (Table S1). The product ion spectrum includes the characteristic hippuric acid fragment at *m/z* 105.0335. Using the same approach, theobromine, cotinine, pantothenic acid, creatinine, 1-methyladenosine, 3-methyladenine, and L-acetylcarnitine could be clearly identified using the SV/CoV reference values from the mix 50 standards (Figure S2, Table S4).

**Fig. 8:**
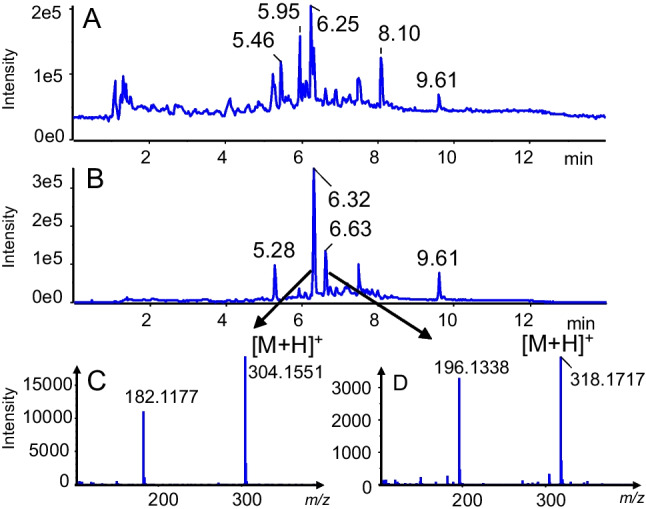
Screening of cocaine, **A** TIC of LC–MS urine sample with DMS in transparent mode, **B** TIC LCxDMS-SWATH-MS experiment, CoV = 8.5 V cocaine and cocaethylene eluting at retention times of 6.32 and 6.63 min respectively. **C** MS2 spectrum of [cocaine + H]^+^ and **D** MS2 spectrum of [cocaethylene + H]^+^ both at CoV + 8.5 V

Figure 8A shows the TIC trace of the experiment corresponding to a CoV of 8 V. The most intense peak at RT = 6.32 min corresponds to cocaine (Fig. 8B) and using mass defect filtering cocaethylene which has an additional peak at RT = 6.63 min (*m/z* 318.170) could be also detected and the MS2 (Fig. 8C) spectrum library search confirmed the identity of the metabolite.

**Fig. 9:**
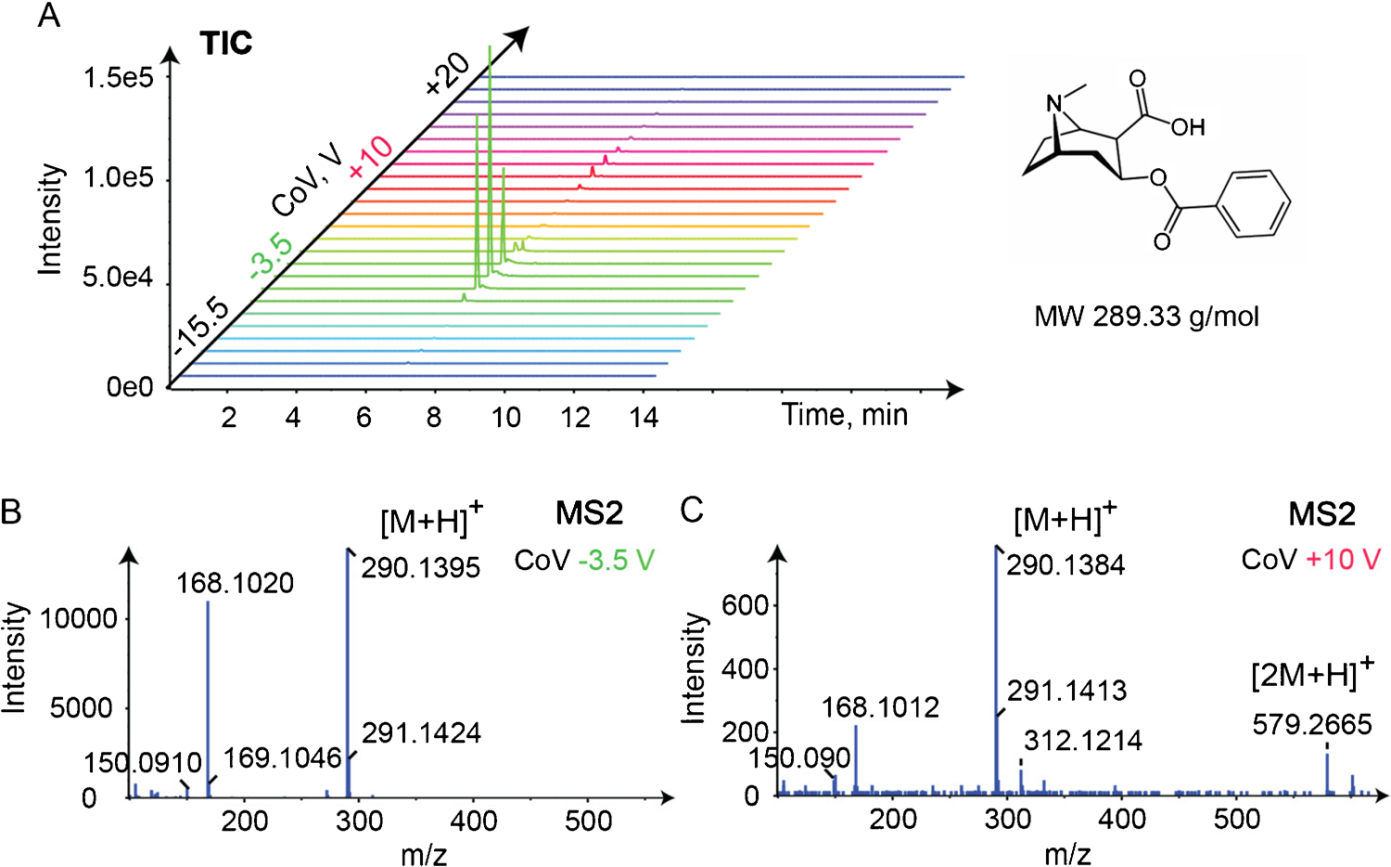
**A** The ionogram for *m/z* 290.1387 in urine sample for LCxDMS-SWATH-MS experiment and scanning CoV from − 50 to + 20 V, by 1.5 V steps with modifier 0.05% mole ratio IPA is represented, **B** MS2 spectrum of [benzoylecgonine + H]^+^ at CoV − 3.5 V, and **C** MS2 spectrum of [2 benzoylecgonine + H]^+^ at CoV + 10 V are represented

Cocaine has two isomeric metabolites norcocaine and benzoylecgonine which can be differentiated by collision-induced dissociation with adequate collision energy settings. Figure 9A shows the ionogram from scanning DMS for *m/z* 290.1387 in the urine sample. Two CoV maxima are detected at CoV − 3.5 V and at + 10 V both at RT = 6.24 min with the limited LC gradient which may suggest the presence of two isomeric metabolites of cocaine, norcocaine, and benzoylecgonine. From MS2 spectra at CoV − 3.5 V (Fig. 9B), benzoylecgonine could be identified by library search at *m/z* 290.1395 and its characteristic fragment at *m/z* 168.1020 and 150.0910 compared to norcocaine with characteristic fragment at *m/z* 168.1020 and 136.0757. The peak corresponding at CoV + 10 V might correspond to its isomer, norcocaine, but based on the MS2 spectrum was identified as benzoylecgonine protonated dimer at *m/z* 579.2665 (Fig. 9C). To mention is also the presence of the sodium adducts.

## Conclusions

In the first part of this work, we investigated the effect in terms of sensitivity and selectivity of five apolar and polar modifiers (1.5% mole ratio cyclohexane, IPA, Tol, ACN, and EtOH) and one binary modifier (0.05% mole ratio IPA) for LCxDMS-MS analysis. 1.5% mole ratio IPA which is one of the most used modifiers achieved the largest peak capacity of 10.3 (largest CoV range) for a mix of 50 analytes representative of human plasma and urine metabolites. Unfortunately, 25 out of 50 metabolites could not be detected anymore being suppressed by IPA. By lowering the concentration of IPA to 0.05% mole ratio by mixing IPA with a non-clustering modifier such as cyclohexane, a reasonable peak capacity of 8.5 is maintained and 90% of analytes are detected, making this binary modifier the most adequate modifier for multidimensional LCxDMS-MS separation. As IPA is miscible with Ch, binary mixtures can also be used with the standard pump. Despite similar peak capacities being calculated for all modifiers investigated, different selectivities (CoV inversion) were observed and were found to be analyte- and modifier-dependent. Metal adducts and multimers showed also different behavior in DMS. While CCS is gas- and analyte-dependent, the selectivity of the DMS can be tuned using polar or apolar modifiers and is as similar as performing normal phase versus reserved-phase chromatography in a multidimensional LCxLC separation. This opens the possibility to collect more information for the analysis of complex samples. The present investigation reports LC–MS analysis in positive mode ESI but similar benefits with different selectivity are expected in negative mode [8]. The CoV at a given SV and for a specific modifier was found to be useful for analyte characterization under different LC conditions and one could consider building reference libraries to facilitate metabolite identification within a study and between studies. The generic conditions for LCxDMS-MS analysis (binary modifier 0.05% IPA, SV 3800 V) were applied as a proof of concept to analyze urine from traffic control samples with a short LC gradient including a large 700 m*/z* Q1 SWATH precursor selection for collision-induced dissociation. The cycle time was 1.4 s for 48 experiments covering a large CoV range of 70 V. One could consider reducing the CoV range and adding two to three additional SWATH windows for improved MS2 performance.

## Supplementary Information

Below is the link to the electronic supplementary material.

Supplementary file1 (PDF 2044 KB)
