# Supplementary material for: Liquid chromatography and differential mobility spectrometry—data-independent mass spectrometry for comprehensive multidimensional separations in metabolomics
Source: Anal Bioanal Chem. 2023 Feb 23;415(10):1905–15. doi: 10.1007/s00216-023-04602-0 (PMC10050028; doi:10.1007/s00216-023-04602-0)
Supplement: Supplementary file 1 — Supplementary file1 (PDF 2044 KB) [file 216_2023_4602_MOESM1_ESM.pdf]

Supplementary Information

Liquid Chromatography and Differential Mobility Spectrometry – Data Independent Mass Spectrometry for Comprehensive Multidimensional Separations in Metabolomics

Lysi Ekmekciu and Gérard Hopfgartner*

Life Sciences Mass Spectrometry, Department of Inorganic and Analytical Chemistry, University of Geneva, 24 Quai Ernest Ansermet, CH-1211 Geneva 4, Switzerland

*corresponding author at e-mail:gerard.hopfgartner@unige.ch

Figure S1: Overlay of XIC DMS chromatogram for mix 50 analytes obtained from LCxDMS-MS experiments with nitrogen and six different modifiers.

Table S1: Analytes and their corresponding stock concentration, solvent and final concentration in mix 50.

Table S2: Annotation of mix 50 analytes with the respective retention time from LC dimension and CoV values from DMS dimension for a chosen SV.

Table S3: Representation of normalization based on peak area values for mix 50 analytes for nitrogen and six modifiers from LCxDMS-MS analysis.

Figure S2: Screening for known analytes from mix 50 from urine samples of traffic control based on SV/CoV/modifier as analyte identifier.

Table S4: CoV values for analytes from mix 50 for 0.05% IPA as modifier and corresponding CoV values of the same analytes in urine from traffic control.

Figure S1: Overlay of XIC DMS Chromatogram for mix 50 analytes obtained from LCxDMS/MS experiments (A) nitrogen (N_2), CoV -15 to +30 V by steps of 1 V, SV 3800 V and for five modifiers at1.5% in mole ratio (B) cyclohexane (C) 2-propanol (IPA), (E) toluene (Tol), (F) acetonitrile (ACN), (G) ethanol (EtOH) and (C) 0.05% mole ratio IPA , CoV -50 to +20 V by steps of 1.5 V, SV 3800 V expect for E) toluene SV 4000 V.

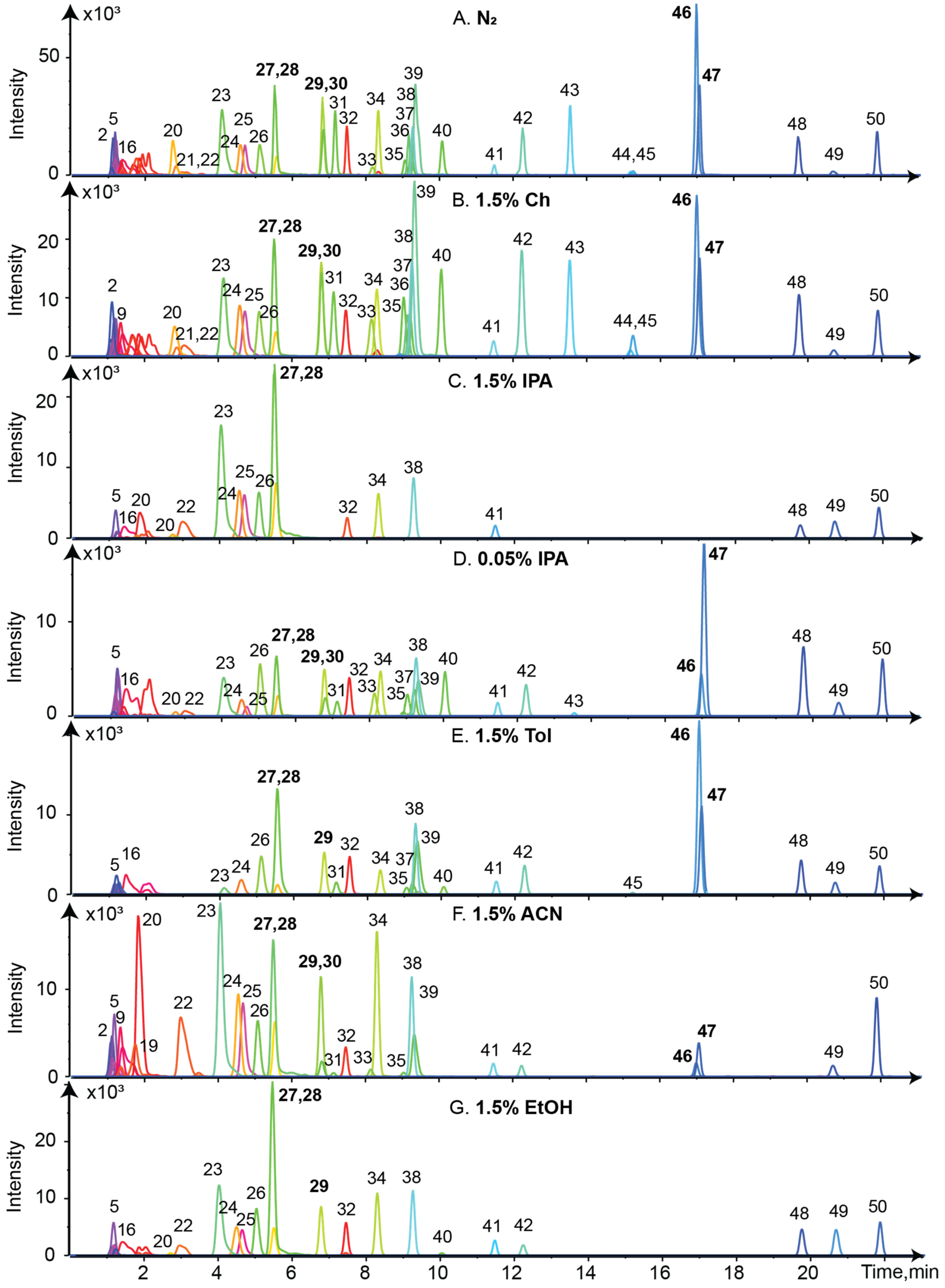

Table S1: Analytes and their corresponding stock concentration, solvent and final concentration in mix 50.

Mix 50	Formula	$[M+H]^+$	Supplier	Eluent (Stock)	Stock solution (µg/ml)	Volume to take (µl)	Conc. in the mix (µg/ml)
L-lysine	$C_6H_{14}N_2O_2$	147.1128	Sigma Aldrich	MeOH/H_2O (1:1)	1000	1000	168
L-Histidine	$C_6H_9N_3O_2$	156.0768	Sigma Aldrich	MeOH/H_2O (1:1)	1000	1000	168
Carnosine	$C_9H_{14}N_4O_3$	227.1139	Brunschwig	MeOH/H_2O (1:1)	1000	250	42
1-methylhistidine	$C_7H_{11}N_3O_2$	170.0924	Santa Cruz Biotechnology	EtOH/H_2O (1:1)	1000	100	17
Glycerophosphocholine	$C_8H_{20}NO_6P$	258.1101	Selleckchem	MeOH	872	229.4	34
Homo-L-arginine	$C_7H_{16}N_4O_2$	189.1346	Selleckchem	MeOH/H_2O (1:1)	1000	200	34
L-Glutamine	$C_5H_{10}N_2O_3$	147.0764	Sigma Aldrich	MeOH/H_2O (1:1)	1000	1000	168
L-carnitine	$C_7H_{15}NO_3$	162.1125	Sigma Aldrich	EtOH/H_2O (1:1)	1000	50	8
N-acetylneuraminic acid	$C_{11}H_{19}NO_9$	310.1133	Selleckchem	MeOH/H_2O (1:1)	1000	1000	168
Creatinine	$C_4H_7N_3O$	114.0662	Alfa Aesar	MeOH/H_2O (1:1)	1000	200	34
Trigonelline	$C_7H_7NO_2$	138.0550	APExBio	MeOH/H_2O (1:1)	1000	100	17
Creatine	$C_4H_9N_3O_2$	132.0768	Sigma Aldrich	MeOH/H_2O (1:1)	1000	250	42
L-Proline	$C_5H_9NO_2$	116.0706	Sigma Aldrich	MeOH	1000	1000	168
Homocitrulline	$C_7H_{15}N_3O_3$	190.1186	Selleckchem	MeOH/H_2O (1:1)	1000	250	42
N-acetylputrescine	$C_6H_{14}N_2O$	131.1179	ABCR	H_2O	1000	200	34
L-acetylcarnitine	$C_9H_{17}NO_4$	204.1230	Santa Cruz Biotechnology	MeOH	1000	50	8
4-guanidinobutanoic acid	$C_5H_{11}N_3O_2$	146.0924	Sigma Aldrich	MeOH/H_2O (1:1)	1000	200	34
3-methyladenine	$C_6H_7N_5$	150.0774	Cayman Chemicals	MeOH/H_2O (1:1)	1000	50	8

Urocanic acid	$C_6H_6N_2O_2$	139.0502	Selleckchem	H_2O	878	1139	168
7-methylguanine	$C_6H_7N_5O$	166.0723	Sigma Aldrich	H_2O	887	230.7	34
Niacinamide	$C_6H_6N_2O$	123.0553	Sigma Aldrich	MeOH	1000	250	42
Tyramine	$C_8H_{11}NO$	138.0913	Sigma Aldrich	MeOH	1000	1000	168
Cotinine	$C_{10}H_{12}N_2O$	177.1023	Sigma Aldrich	EtOH	1000	50	8
Guanosine	$C_{10}H_{13}N_5O_5$	284.0990	Sigma Aldrich	DMSO	1000	1000	168
N-methylnicotinamide	$C_7H_8N_2O$	137.0709	Brunschwig	MeOH	1000	100	17
Cyclic AMP	$C_{10}H_{12}N_5O_6P$	330.0598	MedChemExpress	H_2O	1000	1000	168
Ethenodeoxyadenosine	$C_{12}H_{13}N_5O_3$	276.1091	Santa Cruz Biotechnology	H_2O	1000	200	34
3-chlorotyrosine	$C_9H_{10}ClNO_3$	216.0422	Fluorochem	MeOH/H_2O (1:1)	1000	500	84
Pantothenic acid	$C_9H_{17}NO_5$	220.1180	APExBio	MeOH/H_2O (1:1)	1000	200	34
Acetaminophen	$C_8H_9NO_2$	152.0706	Santa Cruz Biotechnology	MeOH	1000	200	34
Theobromine	$C_7H_8N_4O_2$	181.0720	Sigma Aldrich	H_2O	858	230.4	33
1-methyladenosine	$C_{11}H_{15}N_5O_4$	282.1197	Cayman Chemicals	MeOH/H_2O (1:1)	1000	200	34
Isovalerylglycine	$C_7H_{13}NO_3$	160.0968	Brunschwig	MeOH	1000	250	42
L-Aspartyl-L-phenylalanine	$C_{13}H_{16}N_2O_5$	281.1132	Sigma Aldrich	H_2O	1000	250	42
Hippuric acid	$C_9H_9NO_3$	180.0655	Honeywell Fluka	MeOH	1000	250	42
1,3,7-trimethyluric acid	$C_8H_{10}N_4O_3$	211.0826	Sigma Aldrich	H_2O	849	235.8	34
Chlorogenic acid	$C_{16}H_{18}O_9$	355.1024	APExBio	EtOH/H_2O (1:1)	1000	1000	168
5'-methylthioadenosine	$C_{11}H_{15}N_5O_3S$	298.0968	Sigma Aldrich	EtOH/H_2O (1:1)	1000	200	34
Quinaldic acid	$C_{10}H_7NO_2$	174.0550	Sigma Aldrich	MeOH	1000	200	34
Phenylacetylglycine	$C_{10}H_{11}NO_3$	194.0812	Selleckchem	MeOH	1000	200	34

Riboflavin	$C_{17}H_{20}N_4O_6$	377.1456	Sigma Aldrich	H_2O	329	607.9	34
N-acetyl-L-phenylalanine	$C_{11}H_{13}NO_3$	208.0968	Selleckchem	MeOH	1000	200	34
Indoleacetic acid	$C_{10}H_9NO_2$	176.0706	Selleckchem	MeOH	1000	250	42
Furosemide	$C_{12}H_{11}ClN_2O_5S$	331.0150	Acros Organics	EtOH/H_2O (1:1)	1000	1000	168
Azelaic acid	$C_9H_{16}O_4$	189.1121	Selleckchem	EtOH	1000	250	42
Phloretin	$C_{15}H_{14}O_5$	275.0914	Brunschwig	MeOH	1000	1000	168
Cortisone	$C_{21}H_{28}O_5$	361.2009	Sigma Aldrich	MeOH	1000	100	17
Clotrimazole	$C_{22}H_{17}ClN_2$	345.1153	Cayman Chemicals	EtOH	1000	500	84
Taurocholic acid	$C_{26}H_{45}NO_7S$	516.2990	Santa Cruz Biotechnology	MeOH	1000	1000	168
Glycocholic acid	$C_{26}H_{43}NO_6$	466.3163	Santa Cruz Biotechnology	MeOH	1000	250	42

Table S2: Annotation of mix 50 analytes with the respective retention time from LC dimension and CoV values from DMS dimension for a chosen SV.

Nr.	Mix 50	Formula	RT (min)	CoV (V)						
				SV 3800 V					SV 4000 V	SV 3800 V
				N2	1.5% Ch	1.5% IPA	0.05% IPA	1.5% EtOH	1.5% Tol	1.5% ACN
1	L-lysine	$C_6H_{14}N_2O_2$	1.09	4	3		-31			-39
2	L-Histidine	$C_6H_9N_3O_2$	1.12	1	-1		-38			-46
3	Carnosine	$C_9H_{14}N_4O_3$	1.13	7	6		-16	-41	-20	-29
4	1-methylhistidine	$C_7H_{11}N_3O_2$	1.14	5	3		-2	-42	-7	
5	Glycerophosphocholine	$C_8H_{20}NO_6P$	1.18	9	8	-21	6	-7	3	-31
6	Homo-L-arginine	$C_7H_{16}N_4O_2$	1.19	7	5		-9	-40	-15	-37
7	L-Glutamine	$C_5H_{10}N_2O_3$	1.19	-1	-3		4		8	
8	L-carnitine	$C_7H_{15}NO_3$	1.21	8	6	-39	3	-19	-4	
9	N-acetylneuraminic acid	$C_{11}H_{19}NO_9$	1.25	7	6	-49	-11	-39	-5	-25
10	Creatinine	$C_4H_7N_3O$	1.25	-10	7		5		3	
11	Trigonelline	$C_7H_7NO_2$	1.26	2	-1	-10	-21	-2	-22	
12	Creatine	$C_4H_9N_3O_2$	1.29	-1	-3		4			
13	L-Proline	$C_5H_9NO_2$	1.30	-10	-11					
14	Homocitrulline	$C_7H_{15}N_3O_3$	1.34	4	2	-12	-31	-2	-42	-43
15	N-acetylputrescine	$C_6H_{14}N_2O$	1.34	3	1	-50	-37			-46
16	L-acetylcarnitine	$C_9H_{17}NO_4$	1.40	10	8	-22	6	-8	3	-39
17	4-guanidinobutanoic acid	$C_5H_{11}N_3O_2$	1.69	3	1		10			-47
18	3-methyladenine	$C_6H_7N_5$	1.76	2	1	-41	0	-41	-7	-30
19	Urocanic acid	$C_6H_6N_2O_2$	1.85	-2	-2	-44	4	-50		-19
20	7-methylguanine	$C_6H_7N_5O$	2.76	1	-1	-32	-10	-25	1	
21	Niacinamide	$C_6H_6N_2O$	2.84	-6	-7	-41				
22	Tyramine	$C_8H_{11}NO$	3.01	-4	-5	-42	-34	-42		-27
23	Cotinine	$C_{10}H_{12}N_2O$	4.10	0	-2	-23	-19	-22	-36	-22
24	Guanosine	$C_{10}H_{13}N_5O_5$	4.59	5	4	-32	-22	-30	-33	-21
25	N-methylnicotinamide	$C_7H_8N_2O$	4.71	-2	-4	-36	-36	-35		-38
26	Cyclic AMP	$C_{10}H_{12}N_5O_6P$	5.12	5	5	-36	-9	-28	-13	-21
27	Ethenodeoxyadenosine	$C_{12}H_{13}N_5O_3$	5.53	5	5	-13	-18	-14	-19	-16
28	3-chlorotyrosine	$C_9H_{10}ClNO_3$	5.57	1	0	-36	-21	-37	-27	-24
29	Pantothenic acid	$C_9H_{17}NO_5$	6.82	7	6		-17	-43	-11	-34
30	Acetaminophen	$C_8H_9NO_2$	6.85	0	-2		-36			-29
31	Theobromine	$C_7H_8N_4O_2$	7.16	4	2		-30		-13	-33
32	1-methyladenosine	$C_{11}H_{15}N_5O_4$	7.48	6	6	-34	2	-18	-2	-31
33	Isovalerylglycine	$C_7H_{13}NO_3$	8.18	1	-1		-23			-30
34	L-Aspartyl-L-phenylalanine	$C_{13}H_{16}N_2O_5$	8.33	7	6	-40	-15	-34	-32	-20
35	Hippuric acid	$C_9H_9NO_3$	9.06	2	0		-26		-37	-30
36	1,3,7-trimethyluric acid	$C_8H_{10}N_4O_3$	9.16	3	1					
37	Chlorogenic acid	$C_{16}H_{18}O_9$	9.23	7	6		-11		5	
38	5'-methylthioadenosine	$C_{11}H_{15}N_5O_3S$	9.27	6	5	-24	4	-11	-26	-14
39	Quinaldic acid	$C_{10}H_7NO_2$	9.34	3	1		-37		-16	-48
40	Phenylacetylglycine	$C_{10}H_{11}NO_3$	10.07	4	2		-18	-41	-35	
41	Riboflavin	$C_{17}H_{20}N_4O_6$	11.49	6	6	-24	4	-11	-7	-18
42	N-acetyl-L-phenylalanine	$C_{11}H_{13}NO_3$	12.27	8	6		-18	-39	-27	-23
43	Indoleacetic acid	$C_{10}H_9NO_2$	13.55	4	2		-31			
44	Furosemide	$C_{12}H_{11}ClN_2O_5S$	15.20	14	13				12	
45	Azelaic acid	$C_9H_{16}O_4$	15.27	6	4					
46	Phloretin	$C_{15}H_{14}O_5$	16.99	7	6		-18		-27	-17
47	Cortisone	$C_{21}H_{28}O_5$	17.07	5	5		0		-12	-1
48	Clotrimazole	$C_{22}H_{17}ClN_2$	19.76	6	6	6	6	6	7	
49	Taurocholic acid	$C_{26}H_{45}NO_7S$	20.70	8	7	-13	1	-9	5	-10
50	Glycocholic acid	$C_{26}H_{43}NO_6$	21.90	9	8	-10	3	-6	4	-6
			Analytes	50	50	25	45	29	35	34

Table S3: Representation of normalization based on peak area values for mix 50 analytes for N_2 and six modifiers from LCxDMS/MS analysis.

		Normalization based on peak area						
**Nr.**	**Mix 50**	**N_2**	**1.5% Ch**	**1.5% IPA**	**0.05% IPA**	**1.5% EtOH**	**1.5% Tol**	**1.5% ACN**
1	L-lysine	0.59	0.74		0.12			1.00
2	L-Histidine	1.00	0.84		0.15			0.42
3	Carnosine	1.00	0.57		0.24	0.37	0.33	0.51
4	1-methylhistidine	1.00	0.62		0.69	0.14	0.33	
5	Glycerophosphocholine	1.00	0.53	0.31	0.41	0.47	0.21	0.55
6	Homo-L-arginine	1.00	0.66		0.32	0.36	0.25	0.34
7	L-Glutamine	0.69	1.00		0.06		0.09	
8	L-carnitine	1.00	0.66	0.09	0.44	0.19	0.10	
9	N-acetylneuraminic acid	1.00	0.65	0.18	0.23	0.23	0.30	0.22
10	Creatinine	1.00	0.71		0.49		0.23	
11	Trigonelline	1.00	0.56	0.04	0.06	0.07	0.17	
12	Creatine	1.00	0.82		0.16			
13	L-Proline	1.00	0.84					
14	Homocitrulline	1.00	0.94	0.04	0.22	0.10	0.19	0.54
15	N-acetylputrescine	0.86	1.00	0.02	0.17			0.97
16	L-acetylcarnitine	1.00	0.61	0.27	0.29	0.43	0.49	0.65
17	4-guanidinobutanoic acid	1.00	0.86		0.03			0.32
18	3-methyladenine	1.00	0.63	0.03	0.02	0.03	0.05	0.52
19	Urocanic acid	0.56	0.39	0.37	0.01	0.06		1.00
20	7-methylguanine	1.00	0.47	0.04	0.02	0.03	0.03	
21	Niacinamide	1.00	0.51	0.08				
22	Tyramine	0.09	0.11	0.37	0.04	0.28		1.00
23	Cotinine	1.00	0.54	0.64	0.17	0.51	0.03	0.75
24	Guanosine	1.00	0.78	0.60	0.16	0.49	0.18	0.82
25	N-methylnicotinamide	1.00	0.78	0.61	0.08	0.45		0.80
26	Cyclic AMP	1.00	0.68	0.55	0.47	0.73	0.43	0.55
27	Ethenodeoxyadenosine	1.00	0.67	0.87	0.22	0.87	0.50	0.58
28	3-chlorotyrosine	0.90	0.54	1.00	0.29	0.67	0.16	0.81
29	Pantothenic acid	1.00	0.66		0.21	0.35	0.22	0.47
30	Acetaminophen	1.00	0.89		0.13			0.11
31	Theobromine	1.00	0.55		0.08		0.08	0.02
32	1-methyladenosine	1.00	0.54	0.20	0.34	0.40	0.32	0.22
33	Isovalerylglycine	0.41	1.00		0.36			0.11
34	L-Aspartyl-L-phenylalanine	1.00	0.55	0.29	0.23	0.52	0.14	0.76
35	Hippuric acid	0.52	1.00		0.23		0.09	0.04
36	1,3,7-trimethyluric acid	1.00	0.58					
37	Chlorogenic acid	1.00	0.72		0.30		0.15	
38	5'-methylthioadenosine	0.96	1.00	0.50	0.39	0.68	0.50	0.64
39	Quinaldic acid	1.00	0.88		0.12		0.22	0.16
40	Phenylacetylglycine	0.79	1.00		0.31	0.03	0.06	
41	Riboflavin	1.00	0.82	0.57	0.46	0.86	0.53	0.47
42	N-acetyl-L-phenylalanine	0.90	1.00		0.19	0.10	0.20	0.06
43	Indoleacetic acid	1.00	0.76		0.01			
44	Furosemide	1.00	0.95				0.19	
45	Azelaic acid	0.38	1.00					
46	Phloretin	1.00	0.52		0.09		0.40	0.03
47	Cortisone	1.00	0.57		0.63		0.38	0.13
48	Clotrimazole	1.00	0.82	0.15	0.59	0.37	0.32	
49	Taurocholic acid	0.32	0.22	0.54	0.33	1.00	0.32	0.27
50	Glycocholic acid	1.00	0.56	0.31	0.42	0.42	0.25	0.64
	**Total nb. of analytes :**	**50**	**50**	**25**	**45**	**29**	**35**	**34**

Min	0
Max	1

Where 1 represents the highest peak area per modifier. The normalizatiom based on peak area is calculated as the peak area of the analyte divided by the highest peak area intensity of same analyte per modifier.

Figure S2: Screening for known analytes from mix 50 from urine samples of traffic control based on SV/CoV/modifier as analyte identifier.

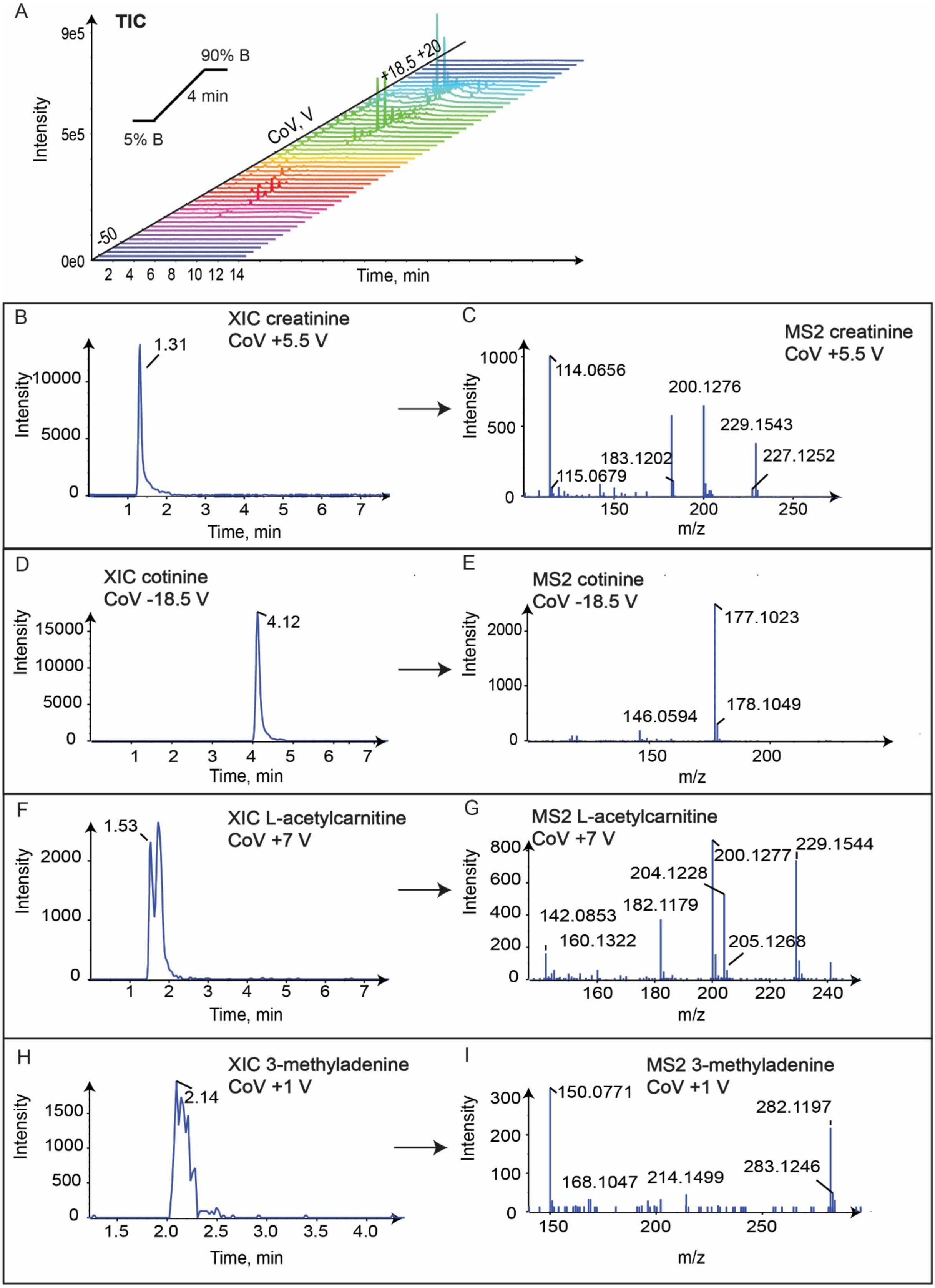

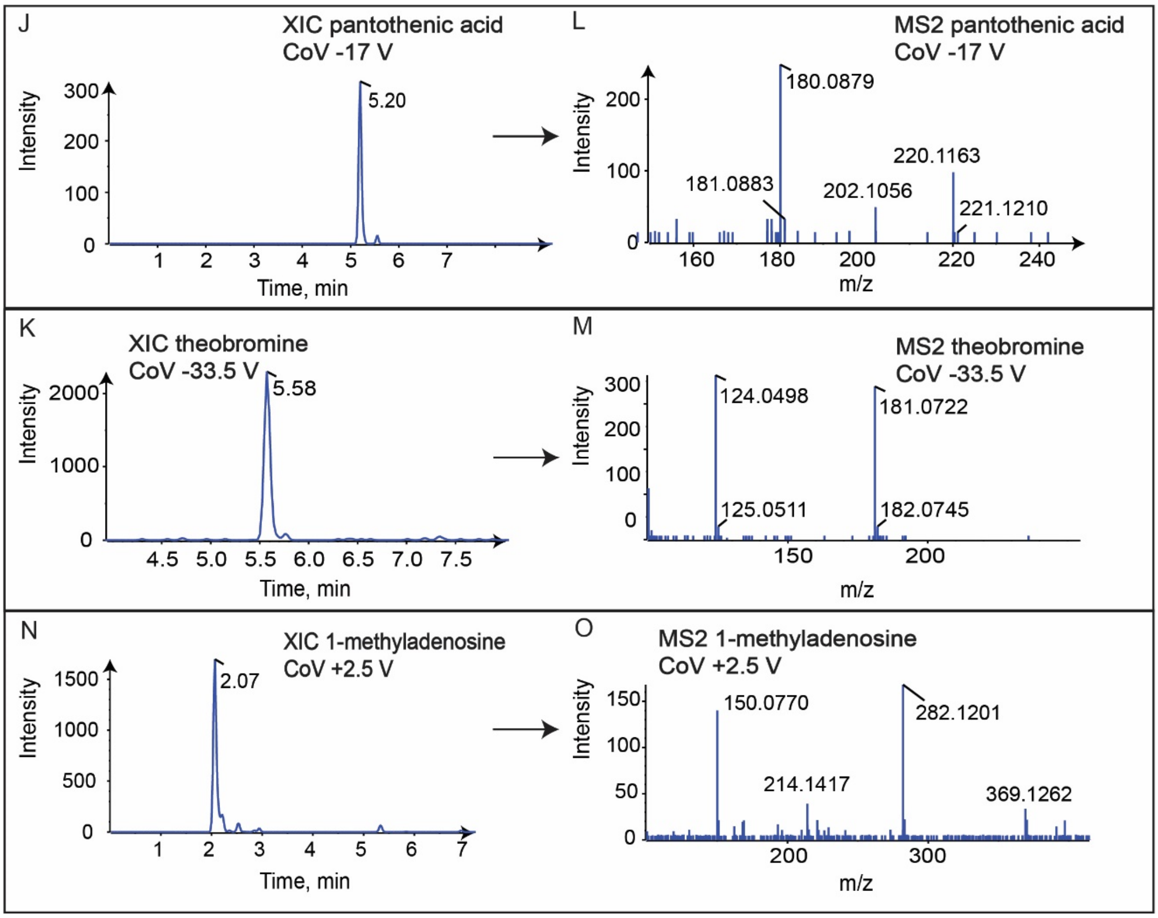

Table S4: CoV values for analytes from mix 50 and CoV values of the same analytes in urine from traffic control with 0.05% IPA as modifier

			CoV for 0.05% IPA	
**Analytes**	**Formula**	**[M+H]+**	**Mix 50**	**Urine**
Creatinine	$C_4H_7N_3O$	114.0662	5	5.5
L-acetylcarnitine	$C_9H_{17}NO_4$	204.1230	6	7
3-methyladenine	$C_6H_7N_5$	150.0774	0	1
Cotinine	$C_{10}H_{12}N_2O$	177.1023	-19	-18.5
Pantothenic acid	$C_9H_{17}NO_5$	220.1180	-17	-17
Theobromine	$C_7H_8N_4O_2$	181.0720	-30	-33.5
1-methyladenosine	$C_{11}H_{15}N_5O_4$	282.1197	2	2.5
Hippuric acid	$C_9H_9NO_3$	180.0655	-26	-26
